# Clinical Efficacy of Blue Light Full Body Irradiation as Treatment Option for Severe Atopic Dermatitis

**DOI:** 10.1371/journal.pone.0020566

**Published:** 2011-06-08

**Authors:** Detlef Becker, Elise Langer, Martin Seemann, Gunda Seemann, Isabel Fell, Joachim Saloga, Stephan Grabbe, Esther von Stebut

**Affiliations:** Department of Dermatology, University Medicine, Johannes Gutenberg University, Mainz, Germany; University of Pittsburgh, United States of America

## Abstract

**Background:**

Therapy of atopic dermatitis (AD) relies on immunosuppression and/or UV irradiation. Here, we assessed clinical efficacy and histopathological alterations induced by blue light-treatment of AD within an observational, non-interventional study.

**Methodology/Principal Findings:**

36 patients with severe, chronic AD resisting long term disease control with local corticosteroids were included. Treatment consisted of one cycle of 5 consecutive blue light-irradiations (28.9 J/cm^2^). Patients were instructed to ask for treatment upon disease exacerbation despite interval therapy with topical corticosteroids. The majority of patients noted first improvements after 2–3 cycles. The EASI score was improved by 41% and 54% after 3 and 6 months, respectively (p≤0.005, and p≤0.002). Significant improvement of pruritus, sleep and life quality was noted especially after 6 months. Also, frequency and intensity of disease exacerbations and the usage of topical corticosteroids was reduced. Finally, immunohistochemistry of skin biopsies obtained at baseline and after 5 and 15 days revealed that, unlike UV light, blue light-treatment did not induce Langerhans cell or T cell depletion from skin.

**Conclusions/Significance:**

Blue light-irradiation may represent a suitable treatment option for AD providing long term control of disease. Future studies with larger patient cohorts within a randomized, placebo-controlled clinical trial are required to confirm this observation.

## Introduction

Atopic dermatitis (AD) is a common inflammatory skin disease affecting approximately 10–15% of all individuals in industrialized countries. A genetic predisposition is well documented. The disease severity can range from infrequent, localized skin affections at predeliction sites to acute exacerbation of the entire skin leading to erythrodermia requiring hospitalization. The patients suffer from significant reductions in their quality of life due to pruritus, sleeplessness and stigmatization [1]–[3].

The pathogenesis of AD is complex. Apart from genetic factors responsible for a predisposition, several alterations of the skin immune system (e.g. differences in the amount of anti-microbial peptides produced by keratinocytes, alterations in the phenotype and function of epidermal dendritic cells with the appearance of normally absent IDEC) and stromal cells (e.g. filaggrin mutation) are important [1]. Acute exacerbation of disease is characterized by a dense infiltrate of the dermis with CD4^+^ T cells showing a Th2-phenotype. Under more chronic conditions, the Th2 predominance is shifted towards a Th1 immune response as shown in atopy patch test experiments [1].

Therapy of AD is based on a regular treatment with basic emollients supplemented with intermittent immunosuppressive therapy of exacerbated skin disease. According to (inter)national guidelines, immunosuppression can be achieved either locally by application of corticosteroid- or calcineurin inhibitor-containing ointments or systemically (e.g. using cyclosporine A, reserved for severe AD) [2], [3]. In addition, UV irradiation (especially UVA1) has proven to be beneficial in those heavily affected patients in whom the above mentioned interval therapy with local immunosuppression is not sufficient for disease control. However, UV radiation has been shown to be carcinogenic and thus, UV phototherapy is usually not recommended as long-term treatment, and for young and immunosuppressed patients [2]–[4].

More recently, it was suggested that blue light irradiation may represent a therapeutic alternative for long term control of AD. Krutmann *et al.* showed that blue light irradiation was found to induce a significant clinical improvement of atopic hand and foot eczema [5]. In addition, anecdotal treatment of several patients with severe AD was reported to be helpful for long-term control of disease. Thus, in the present study we assessed the clinical efficacy and histopathological alterations induced by blue light treatment of patients with severe, chronic AD.

## Materials and Methods

### Patients

Prior to the initiation of the present observational study, the study protocol was approved by the local Ethics Committee of the State Rhineland-Palatinate (Ethik Komission, Landesärztekammer Rheinland-Pfalz), Germany. Between August 1^st^, 2007, and July 31^st^, 2008, all adult patients (≥18 years) who received full body blue light irradiation for AD in our Department were asked to participate in this observational, non-interventional study (n = 36). Inclusion criteria were an age ≥18 years, commitment to non-smoking and the inability to provide long term control of their AD with frequently performed interval therapy using class II/III corticosteroids. All patients included gave written informed consent and showed reliable compliance.

### Irradiation device and treatment protocol

**Figure 1:**
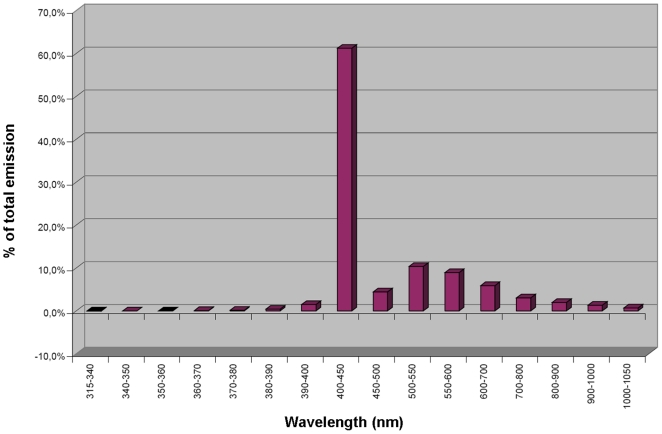
Emission spectrum of the Neuroderm® full body irradiation device.

A photonic irradiation system (Neuroderm®, Spectrometrix Optoelectronic Systems GmbH, Berlin, Germany) was utilized. In this newly-developed full body irradiation device, over 66% of the resulting emission spectrum was between 400 and 500 nm (28.9 J/cm^2^) at a total fluence of 43.7 J/cm^2^ (see Figure 1). A single treatment consisted of a 24 min exposure of each side of the body. The residual UVA emission per treatment was less than 1 J/cm^2^ and the irradiation device therefore fulfilled the criteria for UV-free radiation defined by the International Commission on Non-Ionizing Radiation Protection of the International Radiation Protection Agency (ICNIP/IRA).

Not before 48 hrs after the last treatment within a 5-day treatment cycle, patients were asked to continue treatment with local application of topical corticosteroids, e.g. mometasone 1% cream once daily until their eczema fully disappeared. They were instructed to then discontinue treatment and return to our clinic to receive the next 5-day cycle of irradiation upon reappearance of their AD (flare up).

### Clinical and serological information collected

On day 0 (d0), before treatment was started, clinical and serological parameters were obtained. Similar information and material was obtained on day 15 before the 15^th^ irradiation (d15), and again after 3 and 6 months. On initiation visit, genetic predisposition was judged using the Diepgen Score [6]. Clinical response to treatment was assessed using the eczema area and severity index (EASI) [7], clinical photographs of affected body sites were taken and patients were asked questions about additional treatments, well-being, itching, sleep, and their global assessment of response. In addition, patients were handed the Dermatology Life Quality Index (DLQI) [8] for completion at d0, d15 and after 3 and 6 months.

Routine blood tests involved a differential blood count including eosinophils (normal 0–7%), C-reactive protein (normal <5 mg/l), eosinophilic cationic protein (ECP, normal <24 ng/ml), Total IgE (normal <100 IE/ml). ELISAs specific for human TARC (R&D), IL-4 (ImmunoTools), IL-5 (BD), IL-10 (ImmunoTools), IFNγ (ImmunoTools) and TNFα (ImmunoTools) were performed on serum.

### Skin biopsies

Ethical approval was obtained to analyse the effects of blue light irradiation in skin biopsies of 20 patients before, on day 5 before the last irradiation (end of cycle 1) and on d15 before the 15^th^ irradiation. Informed consent was obtained from 19 patients. Skin biopsies were obtained in local anaesthesia from the same body site from each individual (trunk, legs or arms). Biopsies were generally 0.5×1 cm in size and were fixed in formalin. Sections were stained with H&E to confirm the diagnosis for each patient.

Immunohistochemistry was performed using anti-CD1a (clone M3571, dilution 1∶100, Dako, Hamburg, Germany), HLA-DR (clone LN3, 1∶50), and anti-CD4 (clone 1F6, 1∶40; both Novocastra-Laboratories, Newcastle, England) to quantitate antigen-presenting cells and T cell inflammatory responses, respectively. Stainings were developed using the Chem Mate detection kit from Dako, and analysed using an investigator blinded to the patient's identity using light microscopy.

### Statistical analysis

Statistical comparisons were performed using Statview for Windows and the Students *t* test for unpaired samples.

## Results

### Patient collective

Within a year, 36 adult patients with AD received full body blue light irradiation (compare Fig. 1) within an observational, non-interventional study. The mean age was 36.9 (±2, range 20–57, 15 males/ 21 females), the diagnosis had been confirmed 26 years ago (range 2–48), their mean Diepgen score was 21.5 (±0.9, range 9–32). Due to the observational, non-interventional nature of the study, not all patients responded to follow up requests and were willing to donate blood for further analysis at all time points.

### Clinical response

**Figure 2:**
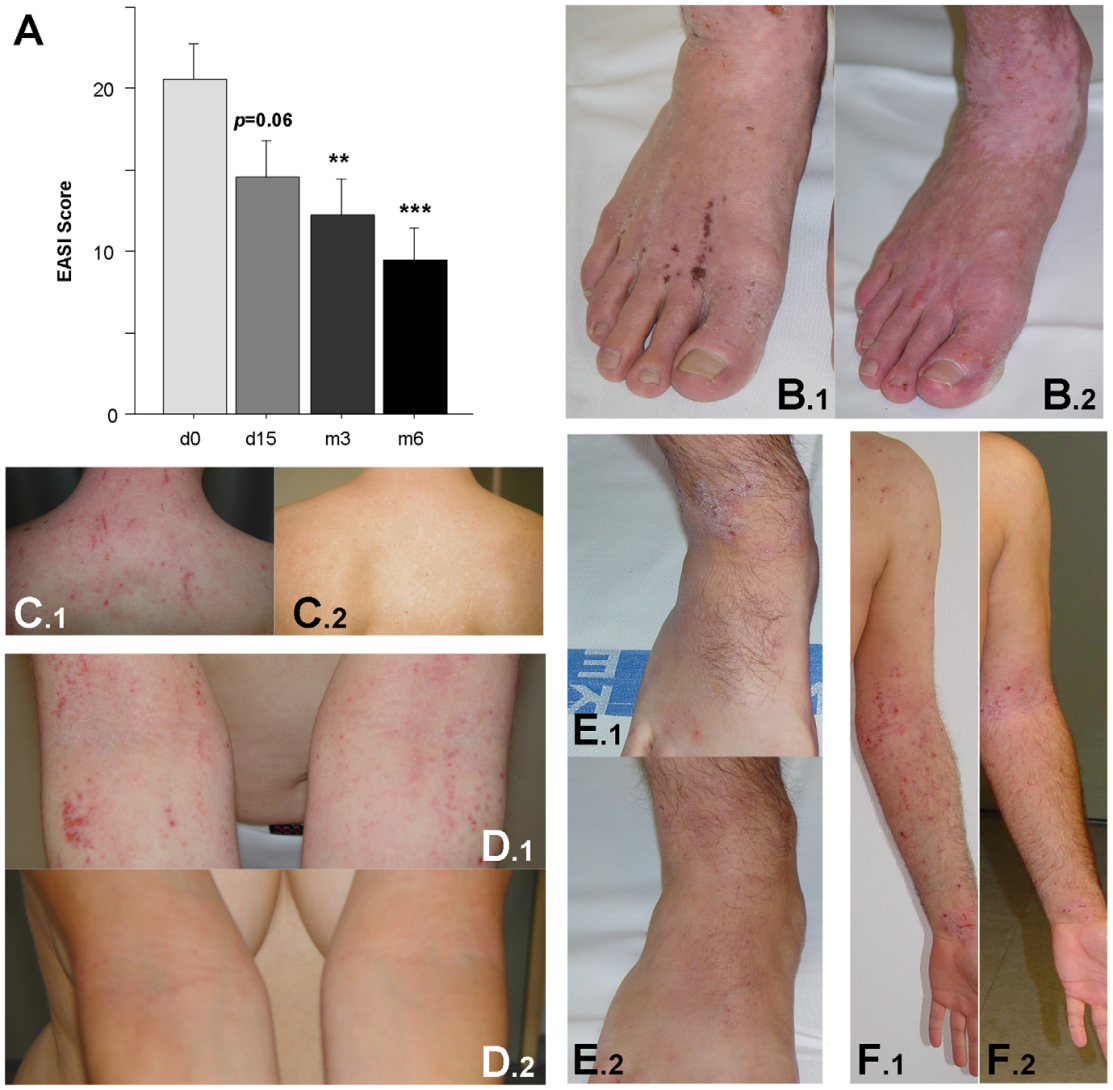
Clinical improvement after blue light irradiation. **A**, EASI assessment of the severity of skin affection was obtained before treatment (d0), on day 15 (d15), and after 3 and 6 months (m3 and m6, respectively). Data are presented as mean±SEM (n≥29, * = p≤0.05, ** = p≤0.005, and *** = p≤0.002). **B**–**F**, Representative clinical pictures from before treatment (.1, d0) and after 6 months (.2). **B+C**: patient #10 – male, 38 years; **D**: patient #15 – female, 41 years; **E+F**: patient #16 – male, 28 years.

The clinical activity of AD was assessed on each visit by a physician. As expected, we noted considerable variations in the baseline level of AD activity. However, the study patient collective consisted of mainly severely affected patients with an EASI [7] of 20.6±2.2 (range 6.8–54) (see Figure 2A). Over time, both the percentage of body surface affected as well as the clinical symptoms erythema, induration, excoriation and lichenification significantly decreased as revealed by an improvement of the EASI: At day 15, 3 and 6 months after treatment initiation, disease severity was decreased by 29%, 41% and 54%, respectively (n≥29, p = 0.06, p≤0.005 and p≤0.002, see Figure 2A).

In addition, on each visit, the patients were photographed to document skin involvement. Figures 2B–F depict the clinical response of 3 different patients. One panel (.1) shows typical AD lesions as head/neck dermatitis, involvement of flexures and feet. The same body sites were photographed after 6 months (panel .2). Notably, skin maceration, erythema and scaling were markedly reduced.

Side effects of blue light irradiation were generally mild and consisted of local redness, warmth and itching of the skin within the first few hours after treatment. Systemic side effects (dizziness or head ache) were reported infrequently and disappeared within a few hours.

**Figure 3:**
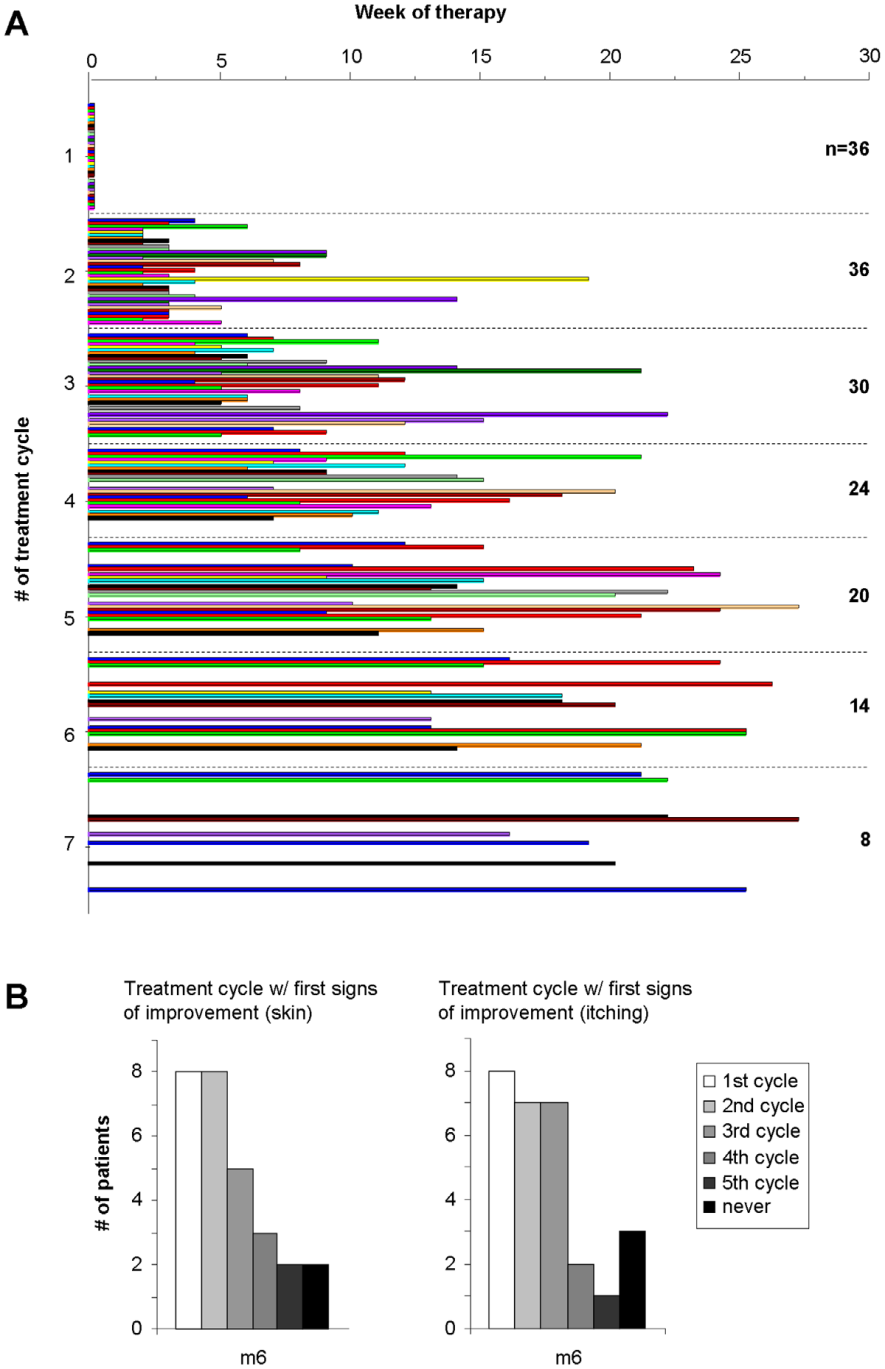
Number of treatments within 6 months and time of first improvement. **A**, For each individual patient (n = 36), the time point at which he/she asked for a subsequent irradiation cycle due to disease exacerbation is depicted. **B+C**, After 6 months, all patients were asked to indicate the irradiation cycle in which they first observed improvement of their skin and/or the itching, respectively.

Next, we assessed the frequency in which the patients received therapy which correlates with the necessity to obtain treatment because of a flare up. As depicted in Figure 3A, all 36 patients asked for a minimum of 2 cycles of irradiation, although each of them after a different time interval. 50% of the patients received at least 5 cycles within 6 months of treatment. Interestingly, the time interval between the last two irradiations increased in the majority of the patients before they did not require additional treatment cycles indirectly indicating that the flare up frequency decreased over time.

Twenty-six of 28 (93%) and 25/28 (89%) of the patients observed improvement of their skin and/or their itching during treatment, respectively; 2 (skin) and 3 (itching) patients did not benefit (see Figure 3B). The majority observed AD resolution as well as a reduction of itching after 2–3 cycles of treatment.

### Effects on quality of life

**Figure 4:**
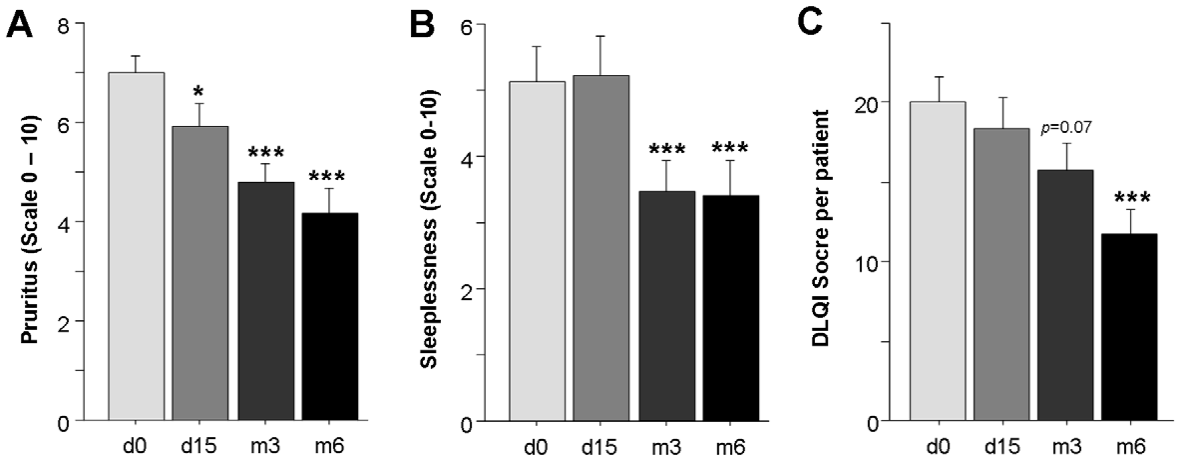
Significant quality of live improvement of blue light-treatment. **A+B**, At the indicated time points, patients were asked to quantify their degree of itching and sleeplessness on an analogous scale from 0 = none to 10 = severe. **C**, Prior to treatment, on day 15 and after 3 and 6 months, each patient completed the Dermatology life quality index (DLQI) consisting of 10 questions which are scored between 0 = not relevant and 4 = very much affected. Data are presented as cumulative data from all 10 questions. **A**–**C**, All data are shown as mean+SEM, * = p≤0.05, and *** = p≤0.002 as compared to d0.

The majority of AD patients suffered from severe itching (7.0±0.3 of maximal 10, mean±SEM, n = 36) and sleeplessness (5.1±0.5 of maximal 10, mean±SEM, n = 36) (see Figure 4A+B). In parallel to disease improvement, the degree of itching was already reduced after 15 days of treatment (p≤0.05), which became highly significant after 3 and 6 months (∼40% reduction, see Figure 4A). In addition, the degree of sleeplessness also decreased significantly over time (see Figure 4B).

Finally, a standardized measure for the life quality was used (see Figure 4C) before treatment (d0), on d15 and after 3 and 6 months [8]. The DLQI consists of 10 questions all scored between 0 ( = not relevant) to 4 ( = very much affected). The DLQI total score significantly improved during treatment (p≤0.005) due to improvements in all categories. Worsening was not observed in any category. Before blue light irradiation, 48% of patients scored the degree by which their skin condition influences their life quality as ‘very much’ or ‘a lot’, but after 6 months of treatment, this percentage was dropped to 21%. At this time, 33% and 46% of the patients stated that their skin influenced their quality of life a little or not at all, respectively.

### Patient assessment

**Figure 5:**
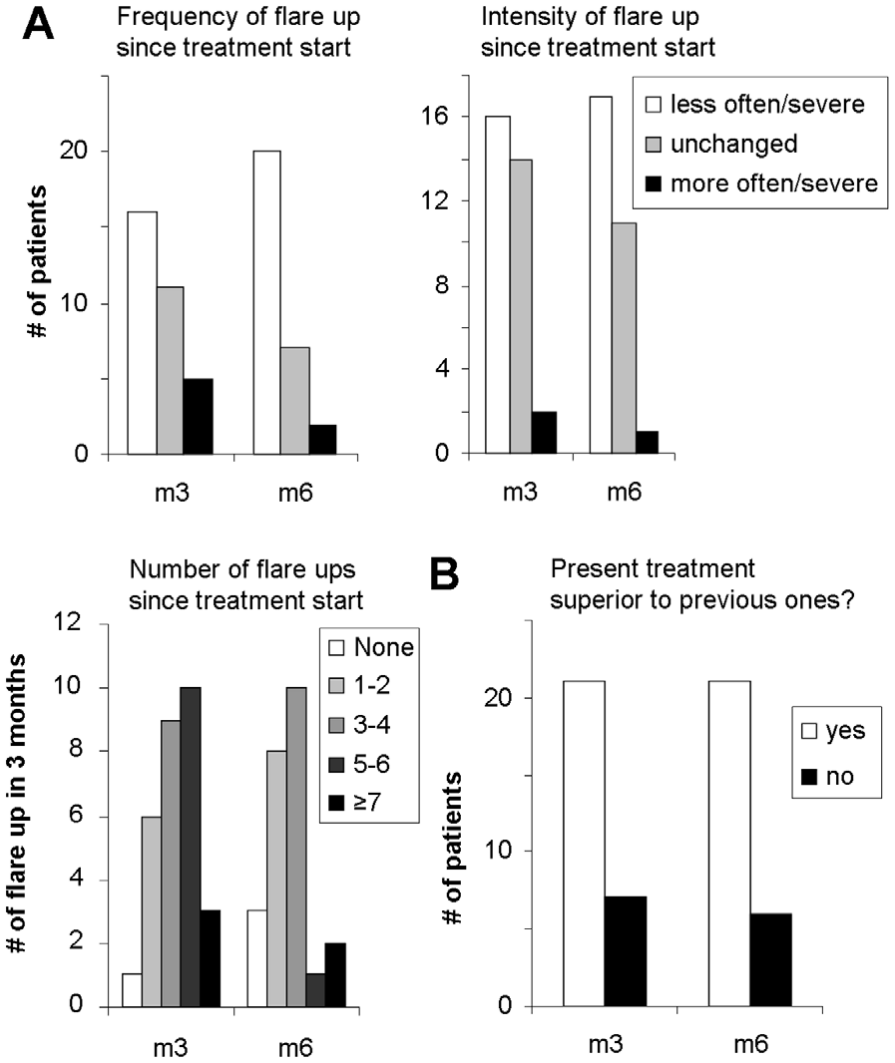
Improvement of severity and frequency of disease exacerbations. **A**, After 3 and 6 months after blue light therapy initiation, patients were asked about their total number of disease exacerbations within the last 3 months, and about the frequency and intensity of these flare ups since treatment start. **B**, The number of patients that judged this treatment superior to prior treatments at 3 and 6 months post treatment initiation is given.

After 3 and 6 months of treatment, patients were asked to judge about the total number, the frequency and intensity of disease exacerbations within the last 3 months (see Figure 5A). All three parameters improved significantly. In addition, the question “Is the present treatment superior to previous ones?” was answered with ‘yes’ in 21 cases (75% and 77%) and with ‘no’ in 7 and 6 cases after 3 and 6 months, respectively (see Figure 5B). Seven of 27 patients observed no changes or an increase in the topical corticosteroid use, whereas 20/27 (74%) used less or much less local immunosuppressants (see Figure 5C). Systemic antihistamines were not routinely taken by all patients; consequently, 7 patients reported to use less systemic antihistamines, whereas 13 observed no change.

### Serological data

Not all patients agreed to give blood upon follow-up visits (64%; n = 23/36 blood samples were analysed). Corresponding with the clinical score, the mean total IgE was significantly elevated in our patient collective at day 0 (7,062±1,999 IE/ml). Over the observation period of 6 months, no alteration of the IgE level was observed (data not shown). CRP levels were not above control levels at any point of time. Eosinophil counts were slightly elevated as compared to controls (9.2±1.2%) and remained the same throughout the observation period. Basic ECP levels were well above normal (56±27 ng/ml). Interestingly, 15 days and after 3 months after irradiation therapy initiation, increased levels of ECP were detected of up to 78 ng/ml, which decreased to baseline by the sixth month. We also assessed the levels of IL-4, IL-5, IL-10, TNFα and thymus and activation regulated chemokine (TARC) in the serum of AD patients. Serum levels of the pro- and anti-inflammatory cytokines TNFα and IL-10 were low and no significant changes observed. IL-4 and IL-5 were negative. Serum TARC was elevated in our AD patient collective at day 0 (1,286±142 ng/ml), but obvious alterations over time were not observed.

### Histological findings

To assess blue light-mediated alterations in the specific skin inflammatory infiltrate of AD patients, we obtained skin biopsies from 19 patients on day 0, day 5 and day 15 post therapy initiation.

First, because mast cells (MC) have been implicated in the pathogenesis of AD, MC numbers in lesional tissue were assessed in Giemsa-stained sections. We observed no alterations of the MC numbers or their degranuation status upon irradiation with blue light (data not shown).

**Figure 6:**
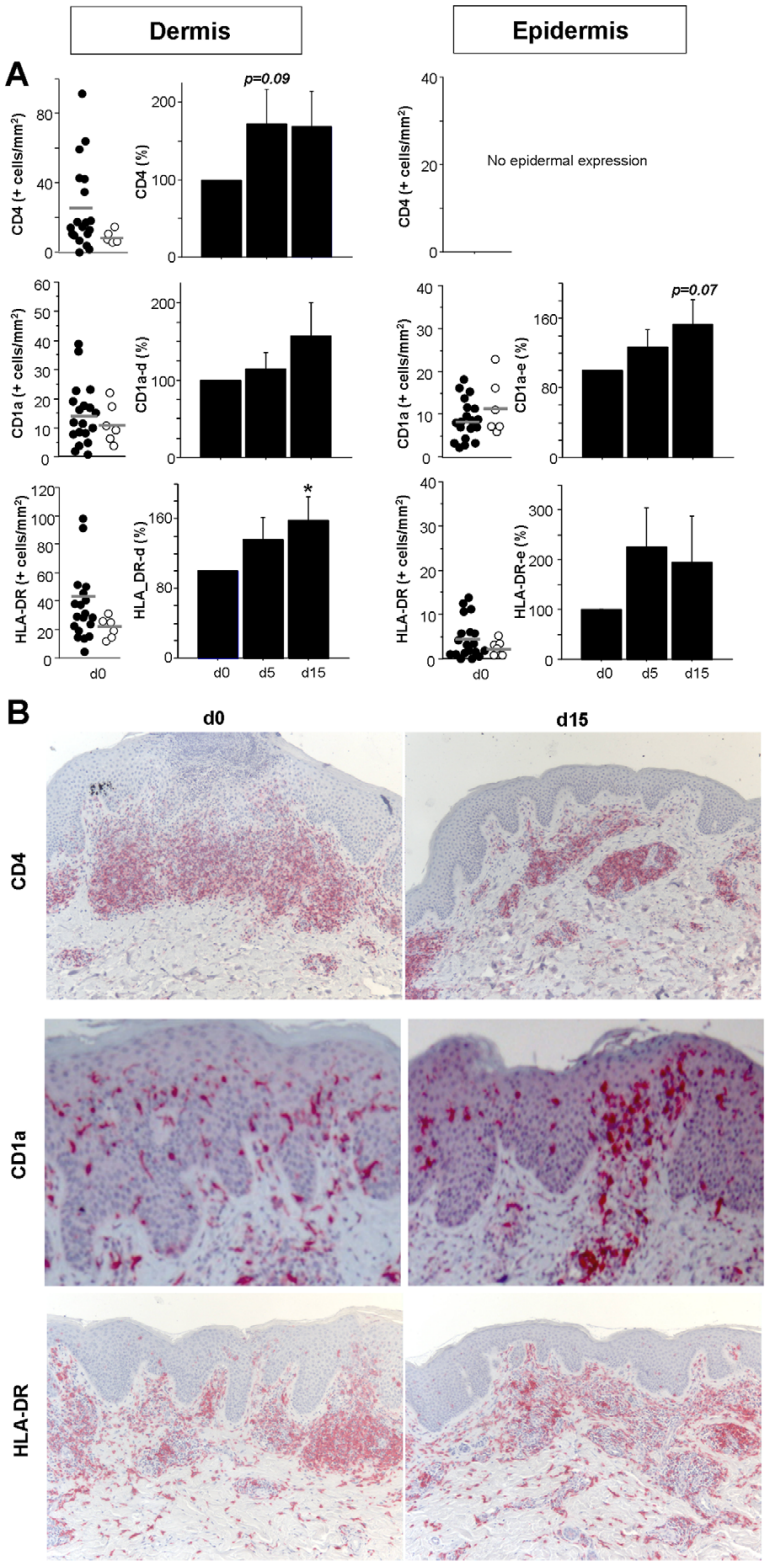
Blue light treatment does not act though similar mechanisms as UV light irradiation. Skin biopsies of AD patients were obtained before treatment (day 0, d0), on day 5 before irradiation and on day 15 before the last irradiation in the third cycle. Formalin-fixed skin was sectioned and immunohistochemistry performed with anti-CD4, anti-CD1a, and anti-HLA-DR. **A**, The number of positive cells/mm^2^ was counted in 5 representative fields per patient both in the epidermis and the dermis. Left panels indicate the mean baseline number for each individual patient (black dots) on d0 with bars showing the mean value of all patients. White dots represent results of healthy control skin from unrelated patients. Right panels contain cumulative data in which the percent change on d5 and d15 to baseline was calculated. Data are shown as mean±SEM (n≥16, * = p≤0.05 as compared to d0). **B**, Representative stainings for CD4, CD1a and HLA-DR are presented for baseline day 0 and day 15 (x100 magnification for CD4 and HLA-DR, x400 for CD1a).

Next, we assessed if blue light therapy resulted in alterations in the inflammation induced by skin-infiltrating T cells (see Figure 6). AD has been shown to be induced by (antigen-specific) CD4^+^ T cells that in the acute phase resemble Th2 cells, whereas chronic AD is characterized by infiltrating Th1 cells. Interestingly, when comparing the number of CD4^+^ cells in skin, unlike UV-mediated effects [8], we did not observe a decrease in the number of T cells infiltrating the skin. In contrast, we observed a relative increase in the number of lymphocytes. In addition, histomorphological signs of lymphocyte apoptosis were not found in H&E stained sections (data not shown).

Finally, antigen presenting cells (APC) were characterized using anti-CD1a (epidermal Langerhans cells (LC), dermal DC), and anti-HLA-DR (see Figure 6). A relative increase in the number of CD1a^+^ DC was observed for both dermal DC as well as Langerhans cells (p = 0.07). Since the staining intensity of the LC for CD1a appeared increased as well (compare Figure 6), we performed additional stainings for HLA-DR, expressed by APC as well as activated T cells (see Figure 6A+B). Both dermal DC and LC expressed more HLA-DR. Thus, in summary, unlike effects of UV, blue light therapy did not lead to a DC depletion from skin.

## Discussion

Atopic dermatitis is a common disease with high socioeconomic impact. In the present study we assessed the efficacy and potential mechanism of action of blue light irradiation therapy of patients with severe, chronic AD. In line with a prior report about treatment of atopic hand- and foot eczema [5], both the clinical response and the patient assessments showed that full body blue light irradiation may serve as attractive treatment alternative for AD providing long term control of disease.

Interestingly, one of the first signs of a clinical response was a decrease in the pruritus as reported by the patients. The overall clinical response was determined as a ∼50% improvement after 6 months as assessed by using the EASI [7]. Other important parameters such as the frequency and intensity of disease exacerbation, topical corticosteroid use and the total quality of life were also improved. Interestingly, the majority of patients observed AD resolution as well as a reduction of their pruritus after 2–3 cycles of treatment (10–15 single irradiations), thus decisions about a continuation of treatment can be made early.

Comparisons with regard to the effectiveness of blue light irradiation as compared to e.g. UV-based treatment regimens are not appropriate. However, it appears that classical first-line treatment of acute AD with UVA1 leads to a more rapid improvement that does not last as long [9]–[13]: improvement based on reductions of the clinical response were ∼30% after 1 week [10], ∼67% and 40% after 3 weeks of cold-light UVA1 and medium-dose UVA1, respectively [11], ∼35% after 3 weeks of medium-dose cold-light UVA1 [12], and ∼32% and 47% after 2 and 4 weeks of bath PUVA compared to 24% and 45% after 2 and 4 weeks of UVB [13]. The improvement observed in our patients who were selected based on their unresponsiveness to classical interval therapy with class II-III topical corticosteroids may thus be comparable to that of classically used UVA1 irradiation [2], [3]. However, improvement of long term disease control and not the ability to provide rapid, acute intervention for disease exacerbation may be the main feature of blue light therapy. The overall effectiveness of UV treatment on AD is well accepted. However, UV is one of the main risk factors for the development of epidermal or melanocytic skin tumors [14]. Thus, the development of UV-free irradiations with proven therapeutic effect is beneficial.

All patients included in the present study were suffering from long-lasting AD with no response to standard interval treatment using topical class II-IV corticosteroid application. Thus, blue light treatment can be considered an “add on” therapy as compared to their prior treatment protocol. However, the contribution of the combinatory therapeutic approach to the overall efficacy of the treatment needs to be evaluated further.

Due to the non-interventional, observational nature of the present study, not all patients agreed to complete all questionnaires and came to follow up assessments. Because of the study design, patients were usually re-evaluated when they experienced a flare up just before they received another cycle of irradiation, so the assessments may in some cases underestimate the overall clinical response. In addition, since treatment cycles are offered only when disease worsening is observed, not all patients received the same amount of irradiation. To better estimate the degree of response in relation to established therapies such as other UV-light treatments, clinical trials with proper control groups need to be conducted.

Using skin biopsies and serological analyses, we have started to investigate the mechanism of action of blue light irradiation in AD. Similar to other treatment modalities, a visible effect of this treatment on serum parameters (i.e. total IgE, ECP, etc.) was not obvious [2], [3]. As described previously, despite the observation that a certain Th1/Th2 ratio in the skin correlates with disease severity, serum levels of pro- and anti-inflammatory cytokines were low and no significant changes observed. Serum TARC was elevated in our AD patient collective prior to the study as reported [15], but alterations were not achieved by therapy.

The blue light-mediated alterations in the inflammatory infiltrate of the skin involved the absence of signs of lymphocyte or DC/LC apoptosis. Our findings are in line with a recent publication demonstrating that application of visible blue light did not cause DNA damage or early photo-ageing; the biological effects observed on normal skin were transient melanogenesis and cellular vacuolization without resulting apoptosis [16]. Thus, the authors concluded that (short-term) utilization of visible blue light in dermatological practice may be safe [16]. Our observations are in stark contrast to the well known effects of UV-light on skin inflammation, which include T cell apoptosis, and depletion of LC from skin [9], [17]. In addition, UV-induced CD4^+^CD25^+^ regulator T cells (Treg) are expanded by UV-exposed cutaneous LC [18]. Both the induction of apoptotic cell death, and the induction of Treg producing IL-10 results in UV-mediated local immunosuppression [9], [19]. In aggregate, even though in retrospect the time points chosen for obtaining the biopsies may not have been optimal and futures studies should attempt to analyse skin responses also at later time points, the effects of blue light on skin inflammation appear to be mediated by a mechanism different from that of UV light. With regard to the molecular targets of blue light irradiation, oxidative stress is potentially relevant. In addition, the degree of DNA damage and/or immune modulatory mechanisms need to be analysed.

In summary, despite this highly selected patient collective with high disease activity and severity, our data strongly suggest that blue light irradiation may represent a suitable treatment option for AD providing long term control of disease. In addition to very few side effects, a good clinical outcome was observed together with a high patient satisfaction. Our data together with more information on a possible mechanism of action will have to be confirmed and extended in a larger patient cohort within a randomized, placebo-controlled clinical trial.
